# 

**Published:** 1909-02-15

**Authors:** 


CONTENTS

Event and Comment - - - - - - - - - 55
The Use of Porcelain in Dentistry, *By Chalmers J. Lyons, D.D.S.* 58
Obituary - - - - - - - - - - - - 84
Bibliography - - - - - - - - - - 86
Indents - - - - - - - - - - 88
Announcements - - - - - - - - - 104

